# Sci-Fin: Visual Mining Spatial and Temporal Behavior Features from Social Media

**DOI:** 10.3390/s16122194

**Published:** 2016-12-20

**Authors:** Jiansu Pu, Zhiyao Teng, Rui Gong, Changjiang Wen, Yang Xu

**Affiliations:** School of Computer Science and Engineering, University of Electronic Science and Technology of China, Chengdu 611731, China; zhiyao.teng@foxmail.com (Z.T.); rui.gong_gr@foxmail.com (R.G.); changjiang.wen@foxmail.com (C.W.); xuyang@uestc.edu.cn (Y.X.)

**Keywords:** visual mining, big data analysis, spatial and temporal behaviors, social media, Internet of things

## Abstract

Check-in records are usually available in social services, which offer us the opportunity to capture and analyze users’ spatial and temporal behaviors. Mining such behavior features is essential to social analysis and business intelligence. However, the complexity and incompleteness of check-in records bring challenges to achieve such a task. Different from the previous work on social behavior analysis, in this paper, we present a visual analytics system, Social Check-in Fingerprinting (Sci-Fin), to facilitate the analysis and visualization of social check-in data. We focus on three major components of user check-in data: location, activity, and profile. Visual fingerprints for location, activity, and profile are designed to intuitively represent the high-dimensional attributes. To visually mine and demonstrate the behavior features, we integrate WorldMapper and Voronoi Treemap into our glyph-like designs. Such visual fingerprint designs offer us the opportunity to summarize the interesting features and patterns from different check-in locations, activities and users (groups). We demonstrate the effectiveness and usability of our system by conducting extensive case studies on real check-in data collected from a popular microblogging service. Interesting findings are reported and discussed at last.

## 1. Introduction

Check-in services such as Foursquare, Facebook Places, and Weibo Locations (Chinese Microblog) are becoming popular and benefiting more and more business applications. Such services allow mobile users to report the places they visit and the activities they are involved in. Collecting check-in data offers us the opportunity to study users’ behaviors which is of high commercial value and has inspired numerous interesting applications, such as geographical space analysis, places of interest study, and personalized recommendation based on social activities. However, social check-in data are usually very noisy, sparse, and contain a multitude of social, spatial, and temporal attributes, which pose the following challenges for analysts.

**Feature missing and discrepancy:** For the checking-in records, users may deliberately hide certain pieces of information due to privacy concerns. To make things worse, some of them deliberately provide false information for various reasons. For example, many people do not use their true locations and event/activity names. The information veracity issue is critical as it may produce large amount of false positive and false negative localization information by analytics purely based on such spatial and temporal attributes [1].

**Ambiguity in behavior features:** The behaviors are usually driven by more than one kind of incentives. For example, users post location or activity information as they are interesting and/or informative. In addition, users may seek friends both for fun and professional experience sharing. To understand the incentives behind the observed behaviors, some guidance should be provided in an explicit or implicit way to identify the correspondence between behaviors and incentives, which, unfortunately, is usually unavailable.

**Deficiency of ground-truth:** The activity involvement process is somewhat subjective. The ground-truth for what activities each user really needs is hard to obtain. Moreover, there can be many exogenous and unpredictable causes for users to check in an activity, which cannot be easily captured from the online data records. Therefore, it is difficult to collect the information which describes in what way the users want to involve in an activity. As one possible solution, a visual mining approach is able to depict the behaviors distribution and features for our investigation to support the next step analysis.

In this paper, we develop a comprehensive visual analytics system, called Social Check-In Fingerprinting (Sci-Fin), to capture user behavior features from their check-in data. The basic idea is to develop visual fingerprints for check-in data, in the form of icons that capture the characteristics of the social, spatial, and temporal attributes of the data. The system allows us to explore three major features contained in the data: location, check-in activity (e.g., dinner or exercise), and profile. We develop three different visual fingerprints: region fingerprint, activity fingerprint, and user fingerprint to capture the characteristics of the space-time features, activity-event features and user-topic features. Our fingerprint designs meet the three critical metrics of an outstanding visualization analysis system, intuitive, compact, and informative, which is a challenging task. To achieve the intuitive requirement, we propose a novel design to incorporate the well-established visualization methods such as WorldMapper [2], Voronoi Treemap, and radial layout. Even though our designs are compact, they are easily recognizable, and can easily be embedded or overlaid into other visualization methods such as graphs, charts, and tables. Our designs are informative as we present various spatial, temporal, and social attributes in one direct display. Thus Sci-Fin facilitates the intuitive comparisons of check-in behaviors at different locations, of different activities, and from different people. The output of Sci-Fin directly assists the complex analytical tasks such as correlation, co-occurrence and anomaly detection involving location, activity, and people. We deploy our system in field to demonstrate the usage of our system with real check-in data from millions of users. Interesting findings at last are obtained and discussed for future research.

To summarize, the contributions of our work are as follows.

- Three novel visual fingerprint designs that capture the characteristics of check-in data from the aspects of space-time features, activity-event features and user-topic features. The designs are intuitive, compact, and informative.
- A comprehensive visual analytics system based on the fingerprint designs to facilitate the analysis of social check-in data and uncover people’s spatio-temporal patterns from the data.
- Field implementation in real social service platform, case studies with real check-in data and interesting findings on users’ check-in behaviors.

The remainder of the paper is organized as follows. Section 2 surveys related work. In Section 3, we introduce our design. In Section 4, the system implementation is described. We report the empirical experiment results in Section 5. Interesting findings and discussions are provided in Section 6. We conclude our work in Section 7.

## 2. Related Work

Emergence of location-based social networks has spawned lasting research on examining the relationship between online world and physical world. For example, [3,4] used mobile phone data set to address two complementary directions of the interplay between humans’ social relationships and their mobility patterns. While Wang et al. [4] demonstrated the possibility of using movement similarity to achieve social link prediction. Sadilek et al. [5] and Backstrom et al. [6] tackled the similar problems, but using online social media data Twitter and Facebook, respectively. We focus on techniques most relevant to our Sci-Fin system, including visualization of social check-in data, icon-based visual design, and spatio-temporal data exploration.

**Visualization of Social Check-in Data:** Since social media are new (Facebook was launched in 2004 and Twitter came along two years later) and check-in services appeared even later (Foursquare is a location-based online social network founded in 2009), there is not yet a large body of academic research on visualization of social check-in data. As check-in data from the public are becoming increasingly available for researchers to analyze, there have been a number of recent works finding new ways to extract various insights on relations between online and offline interactions [7,8], large scale urban dynamics (Noulas et al. [9]; Chang and Sun [10]) and the effects that location technologies have on human behaviors [11,12]. In [13], researchers utilized millions of check-in users to create a dynamic view of a city’s workings and characters. This work proposes to use check-in data to analyze human behaviors in flexible, mobile and fragmented social systems with the weakening of traditional boundaries such as neighborhoods. But we find out that there are few works in the visual mining field. An interactive check-in map provided by Foursqure shows its last 500,000,000 check-ins on a geographic map in an interactive manner and it clearly reveals clusters of people checking into their favorite locations. Another work [14] proposed a hot spot detection method based on frequency and a variation heat map of check-in data from the Chinese Jiepang website and their correlation experiments between the resident population and the number of check-in users in different districts show that the check-in data has a high correlation with the urban economy and population. Kim and Xing [15] visualized brand associations from web community photos. Existing work are usually focused on displaying than analyzing or mining. In our system more advanced visualization techniques have been implemented in a comprehensive study on social check-in data and extracts a deep insight into its spatio-temporal features. Our visual design can simultaneously provide visual analytics from multiple aspects of visualizations of spatial, temporal and multi-dimensional perspectives that are linked together, helping users better explore, compare, and understand evolving multidimensional social check-in data.

**Spatial Temporal Data Analysis and Visualization:** In order to efficiently organise and manage temporal geographic data sets, which are typically available in terms of sampled points, that are associated with additional attributes and with spatial and temporal semantics and to make them visually readable, typical visualization techniques have been developed in this field. These techniques act as a powerful tool to extract implicit knowledge and loosely related information. Andrienko et al. [16] discussed various characteristics of spatio-temporal data and categorized visualization techniques into three major categories, including direct depiction, summarization and pattern extraction. Direct depiction techniques present movement directly. Traditional methods [17] generally plot trajectory paths directly in 2D/3D according to the geographical context. This type of techniques also includes plotting paths as polylines [16] or stacked bands [18], representing origin and destination of trajectories as points [19], and depicting spatial and temporal information together with space time cube [20]. Proximity-based visualization technique [21] was introduced to discover the human behavior patterns by using proximity PCA to transform spatial information into abstract space and display proximity spatial information against time axis. This is sufficient for small amount of trajectories, but when the data set becomes large and complex, visual cluttering and occlusion problems could appear. When handling large and complex datasets, visual clutter is a major defect. Abstraction and aggregation methods are commonly used [22] in these scenarios. Andrienko et al. [23] systematically summarized possible aggregation methods of movement data. Summarization techniques present data based on statistical calculations and concerns changes of information in space and time, so that analysts can get an overall understanding of the tendencies and investigate aggregated patterns. This type of techniques includes density map [24], multivariate glyph [25,26], and flow map [27]. Scheepens et al. [28] presented a density map of vessel movement data by using color to encode temporal dimensions. An integrative approach was employed in [29] by combining self-organizing map (SOM) with a set of interactive visualization tools. They put feature and index images separately into SOM matrix cells to give a combined representation of the spatial, temporal, and attributive (thematic) components of the data. Another data aggregation approach used the predefined areas in [30]. It applied pixel-based visualization to show the aggregated temporal changes to each grid in the Milan map and the spatial evolution of local temporal variables is clearly visible. Summarization techniques has a natural advantage of reducing the hidden uncertainties of data in spatial and temporal coverage [16]. Therefore, we adopt them in our system along with other visualization techniques to provide a novel solution to a comprehensive analysis of spatial temporal features in social media uses. Pattern extraction techniques present extracted patterns of movement to analysts for interpretation and further investigation. Many patterns, such as the interchange pattern [31] and the group movement pattern [32], have been studied. In our paper, we focus on spatial temporal exploration of large-scale check-in data of urban dimensions, including location, activity, and user data and to provide interactive geographic visualization for similarity exploration and pattern extraction in spatial temporal data. In our work, we not only visualize large-scale spatial temporal check-in data sets, but also embed check-in behavior analysis results to digital maps. We apply a multidisciplinary approach to develop a framework for the analysis of massive movement data taking advantage of a synergy of computational, database, and visual techniques. We introduce our framework and demonstrate its effectiveness by examples.

## 3. Visualization Design

In this section we first present several design rationales that guide the development of the fingerprinting of social check-in data. Second, we provide a detailed description of the three-tiered fingerprint visual encoding methodology. Third, we present the advantages of our design, and discuss the limitations.

### 3.1. Social Check-in Data

As one type of crowd-sourcing geographic data, check-in data contain multitudes of social attribute data. Our social check-in data are collected from Sina Weibo which is the first Chinese localized “Tweet” and has now become one of the most famous Chinese social sharing media. Comprising of the tracking of people’s daily lives, check-in data contain daily information such as location, semantics, and behaviors. However, the accuracy of check-in data is low because of the limits of mobile Internet devices. Through the APIs provided by the Sina Open Platform, we obtained the Point of Information (POI) data of all the cities in China by Sina users from October 2011 to November 2012. The check-in data are stored in the MongoDB database in the form of records. Each record registers all the user and POI information. For instance, time, user ID, location ID, location name, location’s longitude and latitude, activity and details of the posts. Although we had the acess to check-in datasets of all cities in China crawled by our collaborator, we are only allowed to demonstrate the results of Beijing and Shanghai.

### 3.2. Task Abstraction

The Sci-Fin system was part of a people’s spatio-temporal feature exploration project initiated by Huawei Ark Lab. This project was aimed at satisfying several real-world requirements for anomaly detection and was supervised by two data mining experts. Regular research discussion meetings with these experts and review meetings with the project sponsor were held. During these meetings, the experts and the review board clarified their requirements and evaluated the prototypes developed for the project. They also provided many constructive suggestions to improve the system. Our system aims to support the analysis of social check-in data for uncovering people’s spatial temporal behaviors. Social check-in data not only have spatial temporal features but also include many other attributes such as POI, detailed geographic location (longitude/latitude), and their post contexts. Based on these features, we extracted the following three important visualization design features to better understand the check-in users’ behaviors.

- **(V1) Space-time related features:** Spatial temporal attributes are important in user behavior analysis. They allow analyzers to capture exactly when and where users check in and what kind of geographic objects surrounding them. Check-in data contain POI and geographic location information with time records. To visualize or analyze such complex features, we set up a region-based visual design which considers the region as a visualization unit. Regions can be constructed politically (partitioned by governments) or by users (mental maps) and cover a certain area in the geographic space and contain a series of POI. Our visual design aims to simultaneously show spatial attributes with temporal information plus other features such as check-in user activities or numbers in such areas.
- **(V2) Activity-event related features:** As well as the check-in users’ location and time, analyzers also need to know what the users are doing in a certain location or at a specific time point. Therefore analyzers can trace the check-in users’ social trends, evaluate the activity/event’s influence (here we can define it as evolution in spatial temporal dimensions), and monitor the events or activities online to detect anomalies or discover any correlation among activities. Our visual design aims to provide analyzers a combined display to integrate spatial changes with temporal information on a specified activity. The number of users can also be visualized and explored in the view.
- **(V3) User-topic related features:** Many powerful new applications can be developed based on the analysis of check-in users behaviors such as real world personalized recommendations. Based on the personalized recommendations, business people or companies can locate potential customers more efficiently. Therefore, it is important for us to design a visual display in the system to show the spatio-temporal evolution of certain groups of users, and together with their contexts to discover their behaviors.

According to our display design V1 to V3, our system supports the following tasks.

- **(T1) Queries related to a region:** Users may ask what kind of check-in activities occur in which region? What is the total number of check-ins for different activities in that area? Does one dominant activity exist (i.e., an activity contributes the majority of check-in records in a region)?
- **(T2) The queries related to an activity:** What is the spatial distribution of the number of check-in users in different regions? Do any routine patterns in the time-related features exist? Hot spot detection and analysis.
- **(T3) Queries related to a certain user group:** Location-based-service; frequently visited places/time/why(activity); What is the favorite time for users’ activity (check-in)? What is the most likely check-in time for users when they play sport? What is the grouping pattern of a group?
- **(T4) Comparisons of two or multiple regions, two or multiple activities, two or multiple users/user groups:** Can we identify the difference among different regions (actually we want to figure out whether different politically constructed regions can be identified by social activities or not)? Does one kind of activity with some patterns in spatial distribution exist (Concentrated distribution or scattered distribution)? Does the spatial or temporal correlation of different activities exist?
- **(T5) Complex analytical tasks like correlation or co-occurrence patterns involving different regions, activities and user groups:** Does similar geographic info lead to similar visual patterns (activity, user number, temporal distribution)? Does any activity have a global influence or just local? Does a similar living neighborhood of different user groups lead to similar behavior (sequence)?

### 3.3. Design Rationals

Motivated by insights from check-in users spatial temporal behaviors and proposed by a social behavior analysis company, we developed this work to enable domain experts or normal users to uncover people’s spatio-temporal patterns from social check-in data visualization, analysis, and comparison. In addition, experts wanted us to use a visualization approach to validate the accuracy of their classification algorithms since their current solution is labeled and checked manually by an analyzer which is at high cost and time consuming. To achieve these two goals, we adopt an approach by developing visual fingerprints for the check-in data to reveal any patterns and display the results. We identified a few key design rationales to follow during the development of our system Sci-Fin.

- **(D1) Social Check-in data’s visual representation should capture the characteristics of the social, spatial, and temporal attributes of the data.** Check-in data contains three major features; location, check-in activity (e.g., dinner or exercise), and user. These three features evolve both in spatial and temporal ways. An effective visual representation must convey the characteristics of check-in behaviors from the three features respectively in a spatio-temporal manner. Fingerprints adhere to this rational by transforming the check-in data into a three-level icon-based visual design integrated with some well-established visualization techniques.
- **(D2) Fingerprints should encode the hierarchical structure among the three major features including location, check-in activity, and users’ information.** Here fingerprints use the well-established visualization Voronoi Treemap to convey this information in a rather compact display space on the screen. Therefore a D1 based system can simultaneously display the spatial temporal information with such hierarchical information and facilitate the comparison since we can put multiple fingerprints together for analysis.
- **(D3) Any representation of social check-in data’s should be intuitive and informative to facilitate the comparison tasks of check-in behaviors at different locations, of different activities, and of different people.** Therefore icon-based glyph design has been proposed since icons can compactly convey a lot of information which only occupies a small area of the display screen. Icons can also be dynamically placed on interesting or important locations for intuitive spatial comparison. In addition, icon-based design can be very flexible in scale and have a better rendering performance than a pixel-based display. We chose a radial layout since the circular shape can convey most information by encoding most of the Voronoi cells in a compact area based on D2. Here we follow the assumption that most temporal comparison tasks will focus on periodic patterns so we chose to use a circular bar chart laying out the icons to encode temporal changes. For possible linear temporal exploration or analysis tasks, we added the ThemeRiver [33] function into our system with its enriched interactions to visually depict the changes in check-in activity strength over time using a river metaphor and provide an overall tendencies for users. In order to discover the correlation between different spatial locations, we use World Mapper distortion techniques to distort spatial changes in the icon center. As maps commonly used in daily life may be very familiar with the regions’ geometric shapes, we can utilize this fact to convey any spatial changes in a small area (icon center) for analyzers to discover any correlation such as co-location activities.
- **(D4) Fingerprints for similar spatio-temporal patterns should appear visually similar while dissimilar patterns should have unique visual features that are easily distinguishable**. Fingerprints should provide at-a-glance representations that allow users to easily determine which region/activity/user (s) are unique and which are in similar shape. This design requirement can be critical for both pattern identification and comparison tasks. Fingerprints satisfy this design guideline by using some well established visualization methods such as WorldMapper, Voronoi Treemap, and radial layout to form the basic components.
- **(D5) The visual representation should allow users to interactively manipulate check-in data analysis results for refinement and further exploration of interesting patterns.** Therefore our system provides a set of enriched interactions such as filtering on spatial and temporal exploration, density display, zoom and pan, activity- based query in different geographic spaces, grouping and highlights.

### 3.4. Visual Encoding

**Figure 1:**
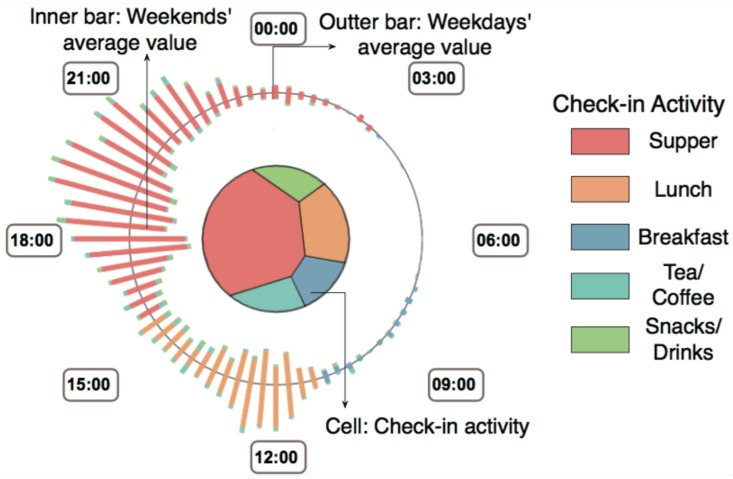
Region-based Fingerp rint of Social Check-in Behaviors. The Voronoi cells inside the icon encode check-in the activity type. The Voronoi cell size encodes the related number of check-in records of that activity. The color is used to categorize the activity type shown in the right part of the figure. The outer bar presents the average weekday value, while the inner bar shows the average weekend value. Each bar is formed by a set of slices of different lengths. One slice on the bar represents one type of activity and its length shows the related number of check-in records.

**Figure 2:**
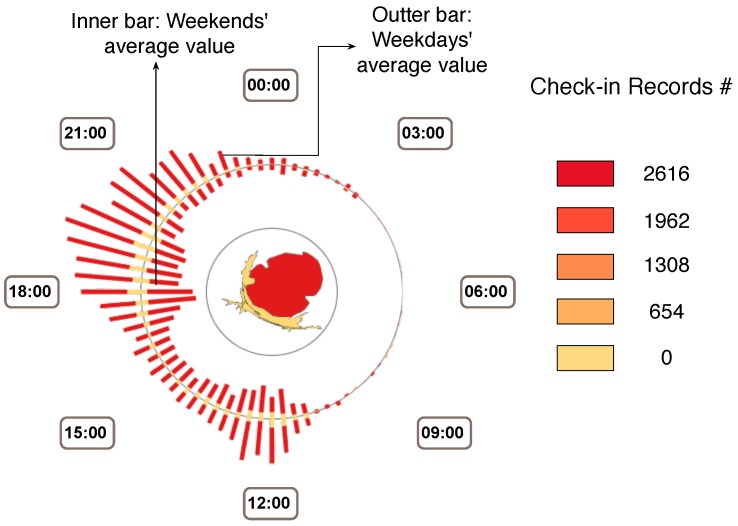
Activity-based Fingerprint on Social Check-in Behaviors. The geometric shapes inside the icon encode selected regions of a city. Here we applied the WorldMapper technique to use space distortion to encode the number of check-ins in that region, while the color represents the density as shown in the right part. The outer bar presents the average weekday value, while the inner bar shows the average weekend value. Each bar is formed by a set of slices of different lengths. One slice on the bar represents one region and its length shows the related number of check-ins.

**Figure 3:**
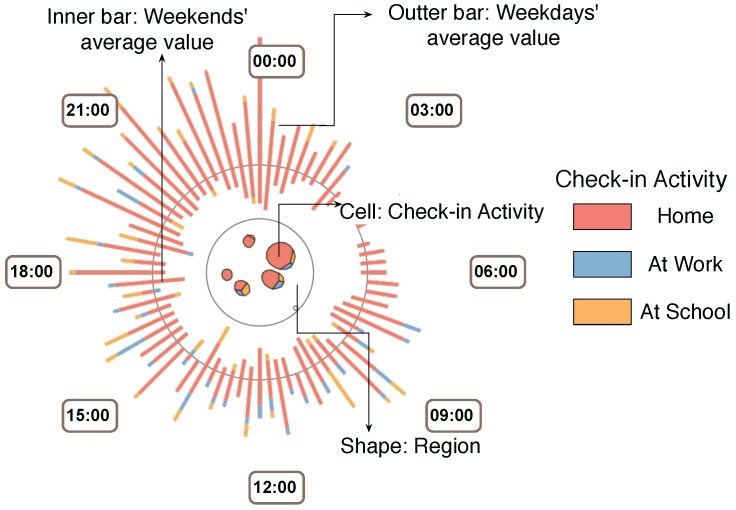
User-based Fingerprint on Social Check-in Behaviors. Inside this icon we use different geometric circles to encode the regions visited by this user group. The Voronoi cells inside the circles encode the type of check-in activity and the size of the Voronoi cell encodes the related number of check- ins of that activity in the region. The color is also used to categorize the activity type shown in the right part of the figure. The outer bar presents the average weekday value, while inner bar shows the average weekend value. Each bar is formed by a set of slices of different lengths. One slice on the bar represents one type of activity and its length shows the related number of check-ins.

Following the design guidelines listed above, we design Sci-Fin as social check-in fingerprinting which represents the evolving features of check-in data in a spatio-temporal way as compact glyphs. As social check-in data contains three major features: location, activity and profile, our icon-based visual design uses a combination of different visualization techniques to convey each major data feature. We provide the details in Figure 1, Figure 2 and Figure 3.

**Region-based Fingerprint on Social Check-in Behaviors.** Each region has one fingerprint on top of it in the geographic map to represent the temporal evolution of the users’ check-in records and activities. In the design we use the voronoi tree map [34] to encode any activity information inside the selected regions.

- **Size.** The size of the Voronoi cells represents one kind check-in activity within one kind of activity.
- **Clock layout.** Then time distribution of the users’ check-in records and activities in the region are encoded along the fingerprints outside the circular broads. We can choose different time scales for display and these displays can be swifted in real-time and smoothly. We provide a 24 h distribution, a week distribution, and a month distribution for the display.
- **Color.** The hue of color is used to represent a check-in activity (see Figure 1).
- **Bar chart.** Each bar chart slice encodes a type of activity. The length of the slice also encodes the number of records.

**Activity-based Fingerprint on Social Check-in Behaviors.** We generated one big fingerprint for each activity extracted from the datasets. In this design, we apply the WorldMapper [2] distortion techniques to represent the spatial changes.

- **Space distortion.** In the center of the activity fingerprint, we display the whole city’s regions’ broads (or either the users can select the interested regions for further exploration). The size of each inside region encodes the number of check-ins for the selected activity in that region.
- **Clock layout.** The time distribution of the users’ check-in records and activities in the region are encoded along the fingerprint’s outside circular broads. We can choose different time scales for the displays and they can be swifted into real-time and smoothly. We provide 24 h distribution, a week distribution, and a month distribution for the display.
- **Color.** Color is used to represent the density of the check-in records (see Figure 2). Both the bar charts on the circle and the distorted regions in the center are colored according to their related check-in number.
- **Bar chart.** Each bar chart slice encodes one region if it has records of selected activities. The length of the slice also encodes the number of records.

**User-based Fingerprint on Social Check-in Behaviors.** In the analysis we generated one big fingerprint for a specified user or user group to meet special scenarios or needs. In this design, we combine the circular shape with a vonoroi treemap to represent the users number and activity changes in the spatial domain.

- **Voronoi cell.** Inside this icon we use circles to encode all the regions visited by this user group from the records. The voronoi cells [35] inside the circles encode check-in activity type. The voronoi cell size encodes the related number of check-in records of that activity in the region.
- **Clock layout.** Time distribution of the users’ check-in records and activities in the region are encoded along the fingerprint’s outside circular broads. We can choose different time scales for display and these displays can be swifted in real-time and smoothly. We provide a 24 h distribution, a week distribution, and a month distribution for the display.
- **Color.** The hue of a color is used to represent check-in activity (see Figure 3).
- **Bar chart.** Each bar is formed by a set of slices of different lengths. One slice on a bar represents one type of activity and its length shows the related number of check-in records.

## 4. System Implementation

### 4.1. System Design and Overview

**Figure 4:**
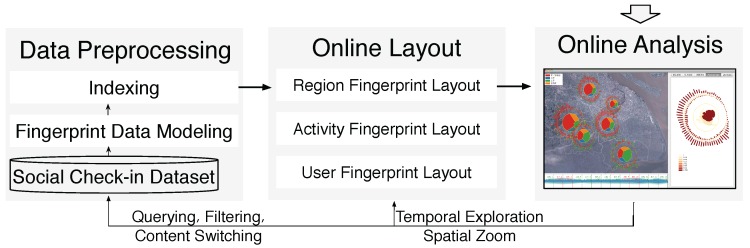
System overview and data processing pipeline. The Sci-Fin system consists of three primary components: (1) a data transformation module; (2) an online layout and rendering module; and (3) a user online analysis and interaction module.

As illustrated in Figure 4, we have developed the Sci-Fin based on task abstraction and design requirements. The system architecture consists of three primary components: (1) the data collection module; (2) the preprocessing module; (3) the analysis module; and (4) the visualization module. First, in the data transformation module, check-in data extracted from the Microblog database is transformed into the fingerprinting data model through feature extraction and activity inference. The transformation process also constructs a set of indices over the data model for online querying. A description of the data model and transformation process can be found in the data model subsection. According to the visual design outlined in Section 4, the online layout and rendering module maps the indexed check-in posts and fingerprints to a Sci-Fin visual display. It also supports user-defined layout input that transforms and renders the raw data immediately into visualizations in real time. Finally, the online analysis and user interaction module enables rich interactions for users to explore check-in data through operations such as filter, query and context switch. These operations feed back into the previous two modules and enable online data exploration for user-driven data exploration.

**Figure 5:**
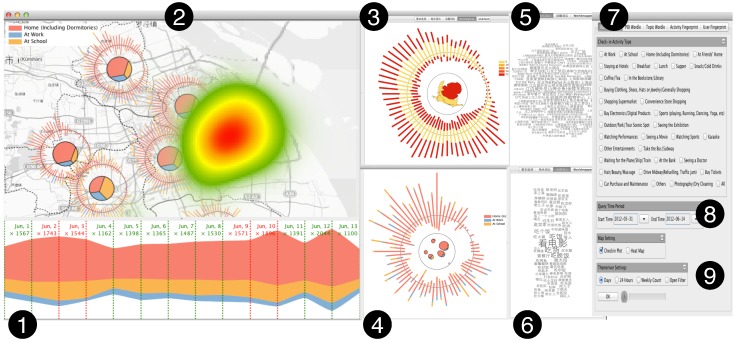
The figure shows two week’s dinning behavior extracted from Sina Weibo (Microblog) check-in dataset in Shanghai. Our system Sci-Fin compressed the whole city’s check-in records related to dinning in real time. In this figure, regions’ fingerprints are placed on map to show their spatio-temporal evolution. The activity fingerprint uses the space distortion inside to represent the records’ geographic distribution. The user fingerprint shows the top 50 users’ spatio-temporal patterns. The numbered annotations from 1 to 9 correspond to major design components and functionality which is described in details in the paper. We developed an interaction “Hightlight” (details can be found in Section 4.3) illustrating all the raw messages that a focused user or users posted during all the activities s/he or they are involved in. And two message views (5 and 6) are proposed to summarize the high frequency keywords extracted from these messages in a Wordle display, showing the overall distribution of the places they uploading records (POI View, 5) and content overview of the messages they posted.

**Figure 6:**
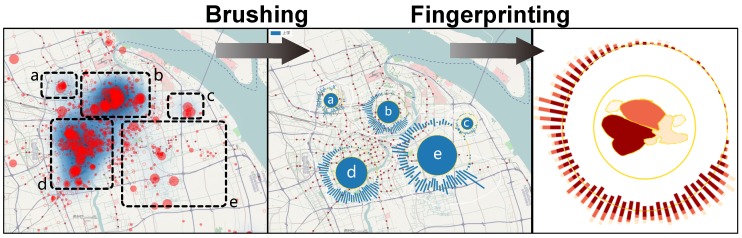
User-controlled Region Partitioning and Fingerprinting. This feature allows users to draw the interested area in any geometric shape and Sci-Fin can handle it as the geometric region input instead of the administration regions constructed by the government. Then Sci-Fin can return the results in real time.

**User Interface.** The user interface of the Sci-Fin system consists of multiple components as illustrated in Figure 5: (1) the Sci-Fin system’s ThemeRiver view that displays the overall temporal trend of the querying check-in data with layers indicating different activities (categorized by color); (2) the geographic map display view and the region fingerprint, check-in posts, and heatmap are also presented in this display; (3) the activity fingerprint view; (4) the user group fingerprint view; (5) the Place of Information (POI) Wordle [36,37,38] display, which use a compact visual form of words extracted from the original check-in records to provide the content overview of visited places; (6) the topic of the check-in data Wordle display; (7) the activity-type-based query box (multiple selection allowed); (8) the query period setting (start time and end time); (9) the ThemeRiver display settings with three different temporal statistical options (24 h, weekly, monthly) and the temporal filter. As expressed in our design guidelines, a key requirement for fingerprint design is that the visualization must allow users to interactively improve the fingerprinting results and progressively refine the analysis results. Our system Sci-Fin incorporates a set of interactive functionalities that support further drill-down to data details along region, activity, and users’ spatial and temporal dimensions (see Figure 6).

### 4.2. Data Model

**Fingerprint Data Model.** We propose a novel spatio-temporal fingerprinting method to discover essential characteristics, or “fingerprints”, by visually exploiting the social check-in data features in spatial temporal means which is inspired by the work in [39,40]. Our fingerprinting method has the following benefits: (a) it leads to spatial temporal data feature extraction; (b) it provides a novel visual structure to answer queries by flexible and dynamic attribute combinations; and (c) it is fast and scalable which is easy to compare. The sophisticated visual form is designed to extract spatial temporal features with multi-dimensions and can well explore the temporal related patterns including periodical and frequent patterns. The “fingerprint” concept used attempts to extract properties of good features by visually/visual analytics to compare trajectories. “Good fingerprints” can also help with anomaly detection and answering similarity queries.

**Figure 7:**
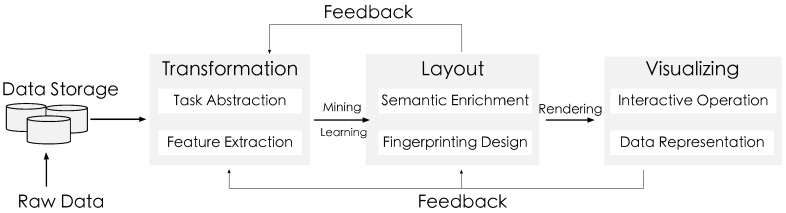
Data model for visual fingerprinting.

Generally, the data model to realize visual fingerprinting can be summarized in terms of four basic stages as follows in Figure 7:

- Data collection and storage.
- Data preprocessing designed to transform the data into understandable information; Extract related features according to actual concrete application tasks; Formulation of expected patterns with mathematic models.
- Enriching data with semantics and knowledge and to design proper layout encoding and rendering algorithms; Display adjustment based on hardware and to produce one or multiple visual structures on the screen.
- Interpretation in the context of the human perceptual and cognitive system and present the proposed visual fingerprints in a intuitive way to better facilitate the comparison and subset of data features exploration.

### 4.3. Interactions

Sci-Fin allows users to interact with the geographic display and to examine relevant data from multiple perspectives. This section discusses all the interactions. As expressed in our design guidelines, a key requirement for fingerprint design is that the visualization must allow users to interactively improve the fingerprinting results and progressively refine the analysis results. Our system Sci-Fin incorporates a set of interactive functionalities that support further drill-down to data details along region, activity, and users’ spatial and temporal dimensions.

**Linking.** The system supports automatic linking among five proposed views not only to support interactive pattern unfolding, but also to facilitate multi-perspective joint analysis. For example, if a specific set of regions on the geographic map or places on the POI Wordle view are selected, the circles in Region View that contains related content and the corresponding visual changes will be highlighted, and other views will be updated accordingly, which enable users to perform a targeted analysis of correlations conveniently.

**Activity-based check-in data query.** Using the Sci-Fin system, users can interactively examine the users’ check-in data online in a certain geographic space by giving an activity type or a set of activity types. They can also query certain activity types by selecting a time period to explore historical check-in data. The querying results can be stored in a local database, which enables further checking and analysis with original records.

**Temporal exploration.** Sci-Fin supports temporal exploration in multiple ways. The temporal filter at the ThemeRiver view allows users to slide back and forth to explore data within different time windows in the history. Users can also specify the time periods for querying by setting start time and end time at the query control panel. Users are allowed to have three views to check the overall check-in data trends by selecting the statistical functionality in the related panel. We provide users three different views of record counts and display them in 24 h circles to draw periodic patterns, records count and display followed the weekly distribution to present the weekly pattern, and intuitively count and display by the days extracted from the querying setting.

**Spatial zoom-in and exploration.** The capability of zooming into the map enables users to explore the check-in data with geographic information, and to further check the density of the check-in data by right-clicking the area and then dragging and releasing it to get the selected area’s local check-in distribution on the screen. The heatmap function is also added into the Sci-Fin to help users spatially explore the check-in data density (See Figure 6).

**Highlighting.** Elements in Sci-Fin, including ThemeRiver layers, cities on the map, users’ check-in posts on the map (red dots), and three fingerprinting icons, can be focused on when the mouse hovers over the elements. When focused on an element, a tooltip containing detailed information such as a posting message, the number of check-in records, the activity name, and the location information are shown. In addition, when one layer theme is highlighted in the ThemeRiver view that all the check-in posts are connected to, the elements focused on are also highlighted on the map through color enhancement. For example, when a check-in post is focused on, it is highlighted in a brighter color (in this case bright yellow).

**Filtering.** Filtering enables analysts to focus on important information, especially when handling data of large scale and with uncertainties. Different types of data filters, including activity filters in ThemeRiver view, temporal filters in 24 h circle/weekly/query period (days), and check-in location filters in the POI Wordle view, are provided in Sci-Fin to interactively eliminate less important information through different views and from different aspects and to facilitate subset comparison.

**User-controlled region partitioning and fingerprinting.** Users can explicitly draw regions in any shape for further analysis on the check-in data. This feature allows users to self-define the geometric region’s input instead of the administration regions constructed by the government. Users can use this function to explore the density distribution of any shape and any piece they like (See Figure 6).

**Filtering.** Once users identify an interesting pattern in the flow view, they can click on the point of interest. Our system will locate the correspond-ing tile and reveal more information to users, such as the graph structure at that time point or related details that may be different from application to application.

## 5. Case Study and Interview

We present four examples to give a comprehensive study of our design. We also deploy our system in Sina Weibo and interview different people to collect the feedback.

### 5.1. Case Studies

#### 5.1.1. Case Study Setup

The experiments are conducted on an Intel Core i5 2 2.3 GHz MacBook Pro with 4 GB memory and an Intel graphics HD 3000 384 Mb board. We employ MongoDB to connect to the database server, Java swing and the Prefuse [41] package to develop visualization modules. The whole system can run on a range of different platforms such as Mac and Windows. The digital map is developed based on an open source online map (OpenStreetMap including bit maps and vectors, describes the objects in the digital map and is updated online). Our system supports interactive real-time visual displays and user interactions.

Our results include four case studies of different areas in the city of Beijing and Shanghai; each reflects one or more of our identified dispersion patterns and our observations on social check-in data. We select these case studies based on the previous task abstraction section (Section 3). In each, we characterize and give a short background of the selected analysis area and describe the patterns that we found from the related fingerprint. Finally we present our expert interview feedback on their findings and suggestions.

#### 5.1.2. Case 1: Region-Based Fingerprinting

**Figure 8:**
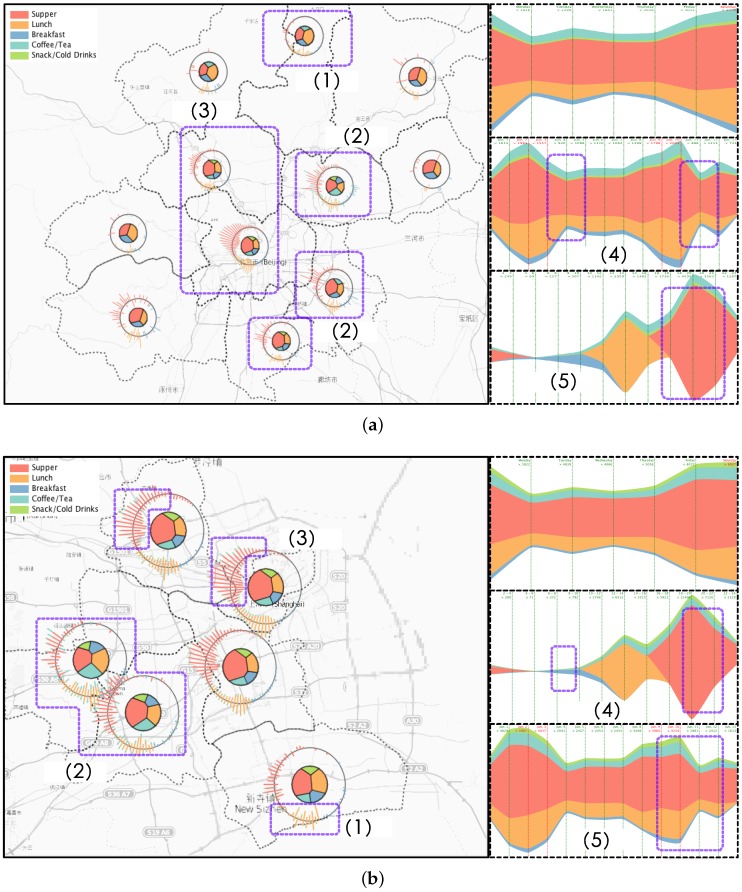
Case 1. The comparison between two regions in the same city and regions from two different cities, show quite different spatial and activity patterns. (**a**) Regional Fingerprinting of Beijing Dinning Behavior. (1) A region’s users choose to have lunch on weekends; (2) Three adjacent regions with more active users on weekdays; (3) A very functional region with all activity types and the popular check-in users; (4) An expected decrease every Monday; (5) Supper for people living in Beijing normally started from 18:00 but slowly decreased by 20:00 then experienced a sharp downturn; (**b**) Regional Fingerprinting of Shanghai Dinning Behavior. (1) A region users choose to have lunch on weekends; (2) Two neighboring regions with similar temporal evolution; (3) A functional region with all activity types and popular check-in users; (4) Supper taken by people living in Shanghai started a little earlier than people in Beijing from 16:00 but dramatically decreased by 20:00; (5) An expected decrease every Monday.

We applied our regional-based fingerprinting design on social check-in records in different regions in the city of Beijing and Shanghai with region boundaries already separated by the government. We are very happy to find that regions in the same city can have different spatial and activity patterns while regions in two different cities can have similar patterns but display unique features. In Figure 8a, we identify six behavior patterns. First in (1) we had found a region in the rural area of Beijing that more users choose to have lunch on weekends rather than weekdays. We also examined two adjacent regions and identified similar patterns. After checking the map we found there are a lot of famous places of interest located in those three regions such as the Great Wall and Wetland parks. Some users also chose to take some tours in their spare time, such as at the weekends, have lunch and return to the city at night. Our second observations was in (2) that there existed three adjacent regions with more active users on weekdays After some field study, we found the region in the top corner is where the Beijing International airport is located. We guess that users may contribute other check-in locations by taking part in activities while they wait for their flights. In the remaining two regions we found some major roads which connected the rural area with the inner city. We guess many people who live rurally, but work in the city center, especially young people trying to save on rent, eat their meals while on route to work. This observation was backed up by the frequent traffic jams we identified as being in these three regions. Observation (3) was that we found most users checked-in to the city center, but more frequently on weekdays than weekends. However, the temporal trends both on weekdays and weekends were nearly the same. We also detected expected temporal changes where the check-in activities decreased every Monday (4). However, we also found that living in Beijing normally had dinner around 18:00, which slowly decreased by 20:00 then reduced sharply to a very low level. In addition, we checked regions in Shanghai of similar design and we found some similar patterns but also identified dispersion patterns. Similarly we found some regions where more users chose to have lunch on weekends in Figure 8b. (1) We had also found some famous places of interest in that region. However, in (2), we identified some different spatial temporal patterns to those in Beijing. Users in these two regions were more likely to have coffee during the day and have supper on weekdays. We checked some background information and the details of some posts. We found there were a lot of middle schools and two famous universities located around the two regions. Therefore we guessed the students were more likely to choose to have lunch at a coffee shop like Starbucks to enjoy a comfortable environment in which to talk with friends or study. In Shanghai we observed the region in (3) attracted most users rather than the city center. As one of the co-authors, who is a data mining expert and worked on the Shanghai taxi data correlation with hotspots, explained that this region is a famous Shanghai hotspot and some famous places like the Bund are located there. In temporal trends, we detected a fall on Mondays as we expect in (5) similar to patterns in Beijing. However, in (4) we identified dinnertime in Shanghai started from 16:00, and had dramatically decreased by 20:00. This is different from patterns detected in Beijing, so we guess two different lifestyles occur because of the two cities’ different environments.

#### 5.1.3. Case 2: Activity-Based Fingerprinting

**Figure 9:**
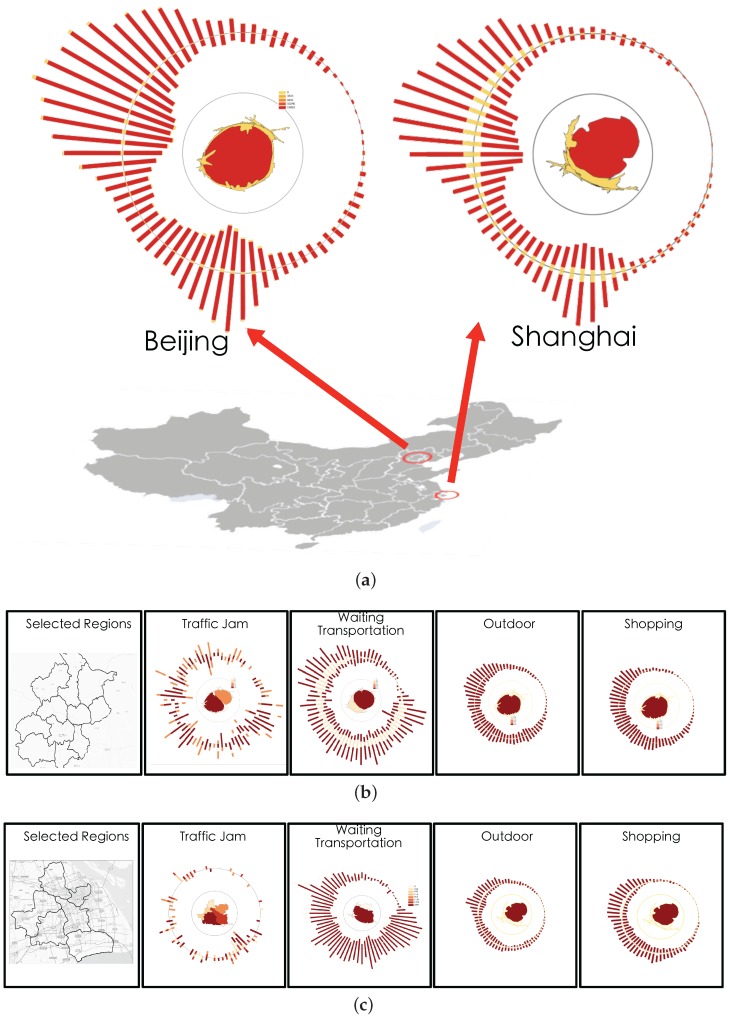
Case 2. The comparison of two activities which show quite different spatial and temporal patterns. (**a**) Two city’s dinning activities fingerprinting comparison. We can easily identify that the temporal trends in two cities differ slightly from each other. The two cities lunch time is closer to 2 p.m. rather than the traditional 12 p.m., however Beijing starts a little earlier and the active period in the night is a little longer than Shanghai; (**b**) Beijing selected activity fingerprinting. Many activities occur in a hot spot, frequent places in geographic spaces or a permanent region. However, we identified that waiting for transportation is an activity that high correlates to the traffic jams in Beijing; (**c**) Shanghai selected activity fingerprinting. We found that many activities occurred in hotspots or frequent places in spatial domains but unlike Beijing, Shanghai’s traffic jams are equally distributed over the whole city.

Based on our T2 task in the previous section, we also provide activity-based fingerprinting results so that analyzers can explore what the users may have been doing at a certain location during a selected time period. Our activity fingerprint design is to visualize one city’s activity spatio-temporal distribution into one compact icon in order for users to compare or search. Figure 9a clearly reveals the difference in the dinning behavior of the people from two big cities in China. We can easily identify that the temporal trends in the two cities slightly differed from each other. Both cities’ lunch times are more likely to start around 2 p.m. rather than the traditional midday. As was normal, Beijing starts a little earlier with its breakfast appearing around 5 a.m. in the morning, while remaining active at night a little longer than Shanghai. For each city we selected four general types of activities including shopping, outdoor pursuits, traffic jams, and waiting for transportation, to generate activity-based fingerprints to display the patterns we found. As shown in Figure 9b we are able to identify the popular check-in activities in Beijing that related to shopping or outdoor activities were located in the center which is inside the fourth major ring road. However, there were two abnormal fingerprints detected. One was the abstraction of the posts when people were waiting for flights, buses, or trains. This fingerprint showed two hot spots in the figure rather just one center. We checked the geographic information and the related post topics then we found the Western Train Station was located at the center of the region while the Beijing International Airport was in the another region. From the visual cue, the region containing the airport contributed to more check-ins than any other places. In addition, when we examined the traffic jam related posts we found it had nearly the same two hot spots as the previous “Waiting for transportation” fingerprint. We suspect the two activities may highly correlate with each other. Figure 9b presents the same four types of activities in Shanghai. Similar to Beijing, the shopping and outdoor activities frequently appeared in one region, but the waiting for transportation differs from Beijing. We identified that the train stations in Shanghai are located near the center of the city but the airport was far from the urban area. From the traffic jam fingerprint we found the activities were equally distributed over the city. Our domain expert explains that this is because Shanghai occupies an area about four times larger than Beijing, therefore major roads in Shanghai are distributed in many regions not just one like Beijing.

#### 5.1.4. Case 3: User-Group-Based Fingerprinting

**Figure 10:**
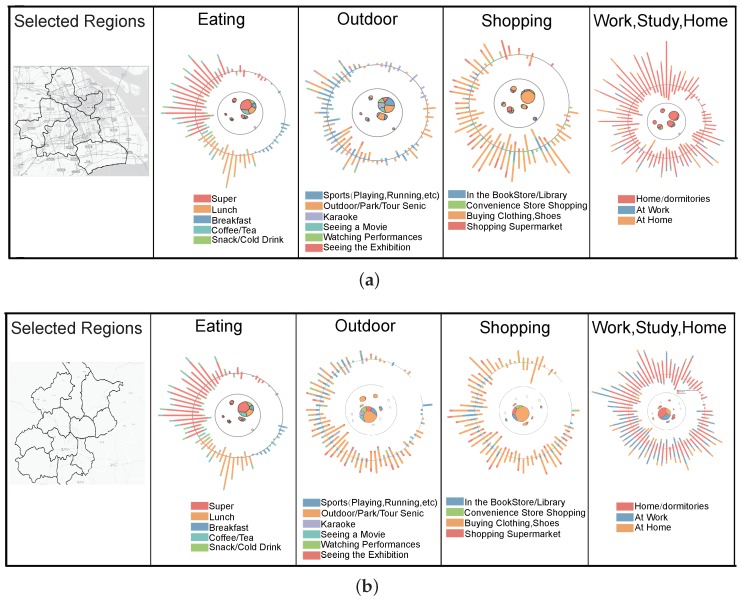
Case 3. The comparison between two user groups shows different spatial, temporal, and activity behaviors. (**a**) User group fingerprinting in Shanghai; (**b**) User group fingerprinting in Beijing.

In this case, we presented certain groups of users spatio-temporal patterns in two big cities and a comparison was also made to infer the difference. Four major types of activities were selected to categorize the user group. We queried the top 50 users rankings in the check-in records posted under dinning, outdoors, shopping, and location (at work, at home, at school) related activities, respectively. Each user group related fingerprint could be found in Figure 10. And we tested our user-group-based fingerprint design in both Shanghai and Beijing. Some interesting patterns emerged. First of all, we detected both cities’ user groups dinning all contributed breakfasts close to 3 a.m.. We thought this might be caused by the activity classification algorithm and backed-up by our data provider. They agreed that these kinds of check- in could be labeled midnight snacks. They marked this problem and decided to improve this in the future. From the eight user groups in the two cities, we found some common characteristics and some dispersion patterns. Outdoor user groups shared the characteristics that users contribute the popular check-in records when they are traveling or taking tours. The second hottest activity in the outdoor group is sports, especially people checking-in at gyms. These two common characteristics may facilitate social networking services to let people know when and where they should go either for sports or to meet friends or people who share common interests. Similarly shopping in malls for clothes or shoes dominated the shopping user group. We think people who go to malls to buy clothes would like to share their states with friends in order to find company or just show off. For Shanghai we find out that users are distributed in five different major regions while in Beijing users choose to stay in the center region. In Beijing we find that schools are aggregated in one region while in Shanghai schools are distributed in multiple regions. Although Beijing’s traffic conditions are in terrible state many people still choose to live in the outside of the center to have lower living cost.

#### 5.1.5. Case 4: Exploration of College Students

**Figure 11:**
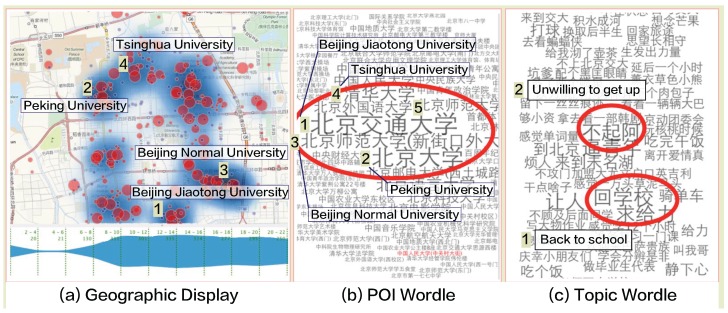
College Students Check-in Records Exploration. (**a**) The geographic display shows the heatmap distribution of check-in records generated by students in Beijing and five universities contributed most check-in records extracted by interaction in POI Wordle view and marked on the map; (**b**) Five universities ranks top five in the records are marked by red circle. The size of each word representing different universities encodes the number of records found in that campus. The top five campus listed in records number order are: (1) Beijiing Jiaotong University; (2) Peking University; (3) Beijing Normal University; (4) Tsinghua University; and (5) Beijing Foreign Studies University; (**c**) The content extracted from users’ original post records. The size of each phrase encodes how many users sharing the idea or action. The top two posts’ keywords shared by most students on campus when semester starts are: (1) Back to school; (2) Unwilling to get up.

In this case, we are particularly interested in the spatio-temporal features of college students especially when each semester starts. As illustrated in Figure 5 two views (5 and 6) are designed to summarize the high frequency keywords extracted from users’ check-in records in a Wordle display. We selected top five campuses with most records generated by users involved in activity type “go to school” from the POI Wordle view (Figure 11b) to analyze. Based on our automatic labeling algorithm provide by our collaborators, the users involved in activity type "go to school" basically could be viewed as students enrolling classes at school. And the top five university picked by POI Wordle view are: (1) Beijiing Jiaotong University, (2) Peking University, (3) Beijing Normal University, (4) Tsinghua University, and (5) Beijing Foreign Studies University listed in records number order. All the places where students check-in are highlighted with words of different size encoding the number of students show the total distribution of the places they reported their locations. According to our interaction design, related spots of five campuses positions are highlighted and marked on the geographic map (Figure 11a). We can see that college students from those five campuses contributed most check-in records and to be very active in the social media than other institutions in Beijing. In order to gain deeper insights, we further check the contents posted by the students in the Topic Wordle view (Figure 11c) and keywords of ideas are extracted from original records and merged. Size of each phrase encodes how popular the idea sharing or spreading among college students. By observing the results from the Wordle display (Figure 11c), we could infer that most students are not recover from holiday status so they do not like to get up so early to go to classes. And the students are also willing to announce they are back campus to their friends.

### 5.2. Interview with Domain Experts

To evaluate the effectiveness of Sci-Fin, other than our collaborators, we demonstrated our system and presented our use cases to three domain experts and conducted one-on-one interviews with them to collect their feedback. Due to the interdisciplinary nature of this research, we choose experts from three relevant professional areas. The first expert (Expert A) is a data mining researcher with expertise in social networks and incomplete data analysis. The second expert (Expert B) is a researcher with expertise in social networks and privacy preserving. The last expert (Expert C) is a researcher with expertise in network system development. Each interview lasted for about an hour.

Interview Methodology. In each of the interview sessions, we start with a tutorial to explain the purpose and provide videos for demonstrating the features of Sci-Fin. After they fully explore the tool’s capabilities, we conduct a semi-structured interview guided by the following two questions:

- Q1 What useful patterns can you observe?
- Q2 Can you conveniently find the problems in the mining algorithms and solve them?

We then ask the experts to use Sci-Fin to visually explore the check-in data. Their feedback is summarized as follows.

#### 5.2.1. Feedback of Interactive Visual Design

All our domain experts confirmed that the system is nicely designed according to the problem domain and the characteristics of check-in data. Expert C thought Sci-Fin is not simply new visualization designs but a combination of visualization techniques together with navigation and interaction techniques to provide a comprehensive system useful to explore spatial temporal features in human behavior from online social media. And they all agreed that this kind of integrated methodology, visualization coupled with flexible navigation, is becoming more and more prevalent to address challenging problems nowadays. Expert B likes our design idea and take it to be an excellent implementation example to display the information of users’ behavior features in multiple visualizations and support flexible exploration schemes through rich interactions. Differing the visualization based on the nature of the information and analytical tasks would greatly enhance users’ understanding. In addition, Expert A considered correlation exploration valuable for practical applications. He further highlighted the design of the region-based fingerprinting and thought this view intuitive and useful while exploring functions of different regions. He also agreed that it could be interesting to extend it to various applications. Meanwhile, all experts believed that the visualizations adopted in our system can be readily comprehended by users with different background.

#### 5.2.2. Applicability and Improvements

All domain experts expressed interests in applying our system to deal with practical problems in their domains. The benefits from the experts can be summarized into two major facts:

- **User Modeling.** The visualization tool helps experts directly uncover people’s spatial-temporal patterns from their check-in data. They can visually observe characteristics such as activity distributions of people along with time and locations. This helps to model users more accurately. For example, they had observed that people in Shanghai have dinner around 1 to 2 h earlier than people in Beijing. This implies that, for the benefit of user modeling, users’ dinnertimes are conditional on the users’ locations. Another important benefit is that experts can get new business models from our visualization tool. For example, the tool can easily show them the time and locations where different people most likely go for physical training. To utilize this information, experts can advise their terminal production group to add a new function in their instant messenger to help people make new friends around them when doing physical training (or more generally, new friends with similar interests).
- **Inspecting the Results of Data Mining Algorithms**
  - **(a) Activity labeling algorithm.** The experts use a rule-based method to label the activity implied in each check-in message. This method uses thousands of expert-defined rules. However, it is not possible for the experts to enumerate all situations that may fall under each activity, or to manually check each rule’s correctness. They have successfully identified abnormal activity patterns with the help of the visualization tool, and corrected our rule base accordingly. For example, the tool shows them that the popular check-in messages that are submitted at Shanghai Customs during working hours were labeled as “working” by their rules, forming a dense sparkle at the location. Upon inspection of those messages, their data engineer found that most were apparently posted by tourists passing through Shanghai Customs. He therefore corrected the original rule condition (which labels a message as “working”) from “people who are in the region of Shanghai Customs during working hours”, to “people who stay in the region of Shanghai Customs during working hours”. This example shows that the visualization tool is able to help the data mining experts continuously improve their activity labeling algorithm.
  - **(b) Activity extraction algorithm.** They use a pattern-based approach to extract “verb-noun” pairs as activity descriptions from check-in messages. The experts then use the visualization tool to study the distribution of activity descriptions in different locations. The tool makes any extraction errors made by their algorithm very obvious. For instance, they noticed two activity descriptions with very high frequencies at a restaurant: “eat (verb) haidi (noun)” and “lao (verb, in English can be explained as catch) hot pot (noun)”. In fact, the correct extraction should be “eat (verb) haidilao hot pot (noun)”. The tool helped experts identify and rectify a lot of such word segmentations and part-of-speech errors, allowing them to generate more accurate extractions.

Involved experts are intrigued by the interactive visualization that fingerprints provide for exploring and comparing check-in users’ spatial temporal behaviors. The experts are attracted to the interactivity and felt that the iconic representations provided significant value. Our data provider and collaborator are also satisfied with the inspecting results for their mining algorithm. They said it effectively improved their efficiency when evaluating their algorithm since they had hired a staff to check all the labeling results with original content. They thought Sci-Fin had showed potential to greatly improve the evaluation efficiency and reduce both the time and cost.

## 6. Discussions and Evaluation

The case studies demonstrate the advantages of Sci-Fin to explore users’ spatial temporal features from online social media. The combination of the different visualizations enables analysts to integrate various information for an analysis from different aspects and at different scales.

**Usability Analysis.** Our activity fingerprint design employs WorldMapper to integrate the spatial changes which provides a number of key advantages. First, the fingerprint designs to capture the characteristics of check-in data from region, activity, and user aspect. The designs are intuitive, compact, and informative and leverage several well established techniques like WorldMapper and Voronoi Treemap; Second, the compact design can encode temporal changes especially periodic patterns with their circular layout and dynamic placement on maps to represent its spatial attributes; Third, the encoding components such as color and shape can be well scaled without any significant loss of information as in [42]. This allows the design to remain effective for both large and small sized icons; Finally, our fingerprint compresses check-in users’ spatial and temporal information with multiple attributes into relatively small icons which can easily be embedded within other visualizations as graphs or tables.

From the interview with experts, we had learned that to cope with threats of contagious diseases is through interviews with people infected, which is neither effective nor efficient. So we were encourage to applied our system with access to real time data to improve the emergency response system for contagious diseases. The experts thought the visual analysis showed a great potential in possible emergency response applications. And Sci-Fci is highly suitable to support business intelligence for example that the advertisers could pre-select the type of customers targeting or serving and Sci-Fci would facilitate him/her to make sensible decisions based on users’ check-in behaviors.

**Discussion of Scale.** Moreover, our system will be faced with scalability issues when the data grows (e.g., when handling data of multiple days, or with large number of regions). To cope with the potential scaling problem, we can extend the filtering of regions or time periods in the geographic view to the whole system. More levels of detail and abstraction can also be introduced to handle this problem. Besides, despite the unprecedented coverage and granularity, we acknowledge that the information derived may underestimate the full repertoire of user behavior features in social media at the spatial and temporal scale considered. Nonetheless, this issue is common to all existing works for analyzing human behaviors at a fine spatial scale. In particular, the number of activity types in a region icon that can be visualized at the same one time is limited because each icon must be represented by a unique easily distinguishable color. For example the original activity type can be up to over 200, the color cannot solve this problem. To address this problem, filtering or clustering/grouping in activity types can be used to reduce the visual clutter in future analysis. Another challenge is that it can be hard for us to visualize too many spatial changes in information in such a compact area when spatial levels increase to a very high level. For example if we want to compare one activity over all the cities in China (more than 650) using our activity fingerprint at the same time, the regions inside will be compressed to a very small size, from which the spatial information inside the icon will get lost due to severe space distortion. In addition, the user icon suffers the same problem. However, we believe these limitations are a reasonable trade-off for the benefit of them intuitively representing high-dimensional, spatio-temporal attributes of check-in data using small, compact icons for facilitating easy comparison.

Our research is still in progress. Weaknesses have been observed and will be addressed in the future. First, our system focuses on visualization techniques and lacks sufficient support for automatic analysis. In many cases, we have to examine data manually with our system to discover patterns, which is like to find a needle in a haystack. If more advanced data mining techniques are incorporated, for example, by recognizing people’s routine behavior patterns to identify regions for home or work, Sci-Fin will be capable of performing more sophisticated analytical tasks. Second, we use color intensity to encode the different activity types extracted from the original dataset in our main display and the user group fingerprint view (see parts 2 and 4 in Figure 5) respectively, but users can only distinguish no more than seven different activity types efficiently due to the restrain by human nature. Thus, more detailed information should be explored through interactions supported by our system. We should adopted more designs and automatic methods together to better present and explore the correlation between multiple activity types. Third, to get the full potential of our proposed fingerprinting design, more advanced correlation metrics and shape transformation algorithms should be explored.

**Figure 12:**
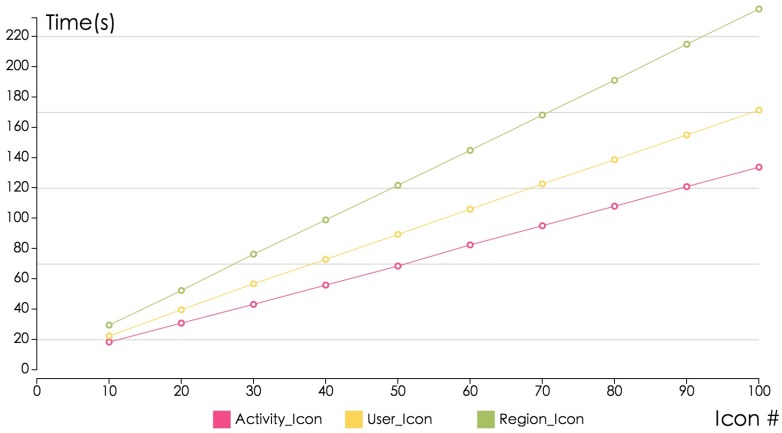
Performance Evaluation. The horizontal axis represents the number of icons generated by our Sci-Fin system. The vertical axis shows the related rendering time and the unit is milliseconds (ms).

Our system is developed based on the visual analytics motivation, thus it is hard to evaluate our design by traditional system and data mining evaluation metrics. Our system’s accuracy is high since it utilizes a human analysts’ intelligence to make the decisions. Analysts can refine or re-tune their results in an iterative way with the help of rich user interactions, so they can achieve satisfying results by progressively improving the parameters. The time cost for our data operations in the database is rather high but the visualization response time is acceptable with the filtering techniques and smooth interaction. We use the 11,160,893 records collected from Beijing and Shanghai to perform our evaluation. We can apply our system to bigger datasets and achieve approximately the same results in real time (See Figure 12). Our system’s performance is limited by the database access and response time. The visualization processing time cost is only 15% of the database access and response time.

## 7. Conclusions

In this paper, we have presented an interactive visual analytics system, Social Check-in Fingerprinting (Sci-Fin), for facilitating the analysis and visualization of social check-in data. A new integrated fingerprinting design of people’s spatio-temporal behaviors has been elaborated to three major components in the check-in data: location, activity, and profile. The behavior fingerprinting design can intuitively represent high-dimensional, spatio-temporal attributes related to these components. In the location (space-time) view of our system, the global temporal changes in spatial evolution are presented to users and can be interactively explored. The activity-event view shows check-in records change to the geographic spaces of specific activities or events assisting with the temporal changes. The user-topic view uses a combinational visualization method to track individual sequences of significant users or groups. Furthermore, the three views integrate important statistical and historical information related to check-in behaviors, which illustrate temporal changes of the users’ behaviors. Some well established visualizations like WorldMapper and Voronoi Treemap are integrated into our glyph-like designs. The visual fingerprint designs allow easy comparison of different check-in locations, different activities, and different user groups. They can also be conveniently overlaid into maps and embedded into graphs and charts. We find that this design is flexible in scale and is compatible to integrate into graphs or tables for assisting statistical analysis. We test our system on a real-life Sina Weibo dataset collected from millions of users and obtained some interesting findings. The experimental results confirm the effectiveness and efficiency of the proposed visual analysis method. The analysis of the results also shows that our system is capable of effectively comparing and analyzing complex social spatio-temporal patterns.

## Supplementary Materials

The following are available online at http://www.mdpi.com/1424-8220/16/12/2194/s1, Video S1: Sci-FinSystem-BasicFunctions, Video S2: Sci-FinSystem-Interaction.
